# Ultrafast Spin Dynamics and Photoinduced Insulator-to-Metal Transition in α-RuCl_3_

**DOI:** 10.1021/acs.nanolett.3c02668

**Published:** 2023-09-11

**Authors:** Jin Zhang, Nicolas Tancogne-Dejean, Lede Xian, Emil Viñas Boström, Martin Claassen, Dante M. Kennes, Angel Rubio

**Affiliations:** † Max Planck Institute for the Structure and Dynamics of Matter and Center for Free-Electron Laser Science, Luruper Chaussee 149, 22761 Hamburg, Germany; ‡ Center for Computational Quantum Physics (CCQ), The Flatiron Institute, 162 Fifth avenue, New York, New York 10010, United States; § Department of Physics and Astronomy, University of Pennsylvania, Philadelphia, Pennsylvania 19104, United States; ∥ Institut für Theorie der Statistischen Physik, RWTH Aachen University and JARA-Fundamentals of Future Information Technology, 52056 Aachen, Germany; ⊥ Nano-Bio Spectroscopy Group, Universidad del País Vasco, 20018 San Sebastián, Spain; # Center for Computational Quantum Physics (CCQ), The Flatiron Institute, 162 Fifth Avenue, New York, New York 10010, United States; ¶ Songshan Lake Materials Laboratory, Dongguan 523808, China

**Keywords:** ultrafast spin dynamics, insulator-to-metal transition, α-RuCl_3_, two-dimensional magnets, TDDFT

## Abstract

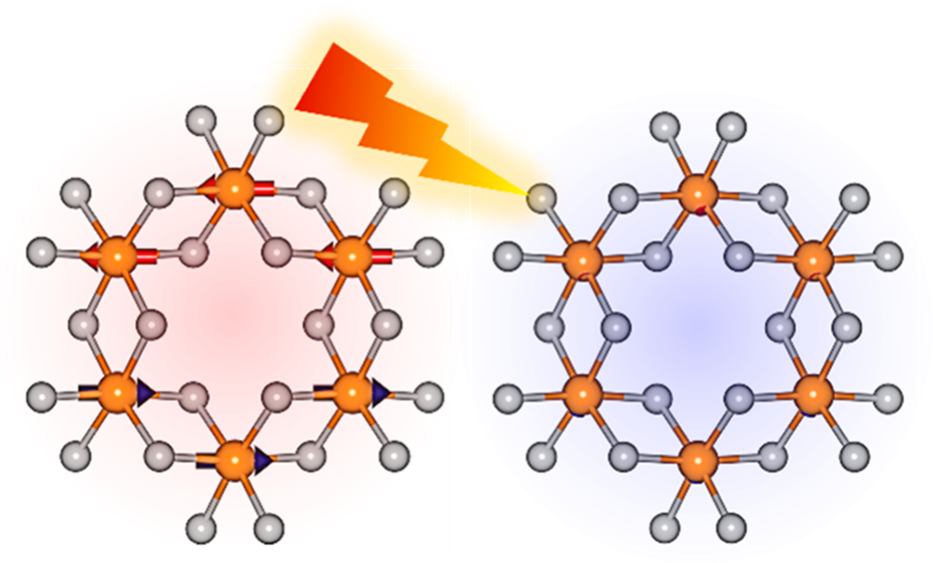

Laser-induced ultrafast demagnetization is a phenomenon of utmost interest and attracts significant attention because it enables potential applications in ultrafast optoelectronics and spintronics. As a spin–orbit coupling assisted magnetic insulator, α-RuCl_3_ provides an attractive platform to explore the physics of electronic correlations and unconventional magnetism. Using time-dependent density functional theory, we explore the ultrafast laser-induced dynamics of the electronic and magnetic structures in α-RuCl_3_. Our study unveils that laser pulses can introduce ultrafast demagnetizations, accompanied by an out-of-equilibrium insulator-to-metal transition in a few tens of femtoseconds. The spin response significantly depends on the laser wavelength and polarization on account of the electron correlations, band renormalizations, and charge redistributions. These findings provide physical insights into the coupling between the electronic and magnetic degrees of freedom in α-RuCl_3_ and shed light on suppressing the long-range magnetic orders and reaching a proximate spin liquid phase for two-dimensional magnets on an ultrafast time scale.

In correlated materials, microscopic degrees of freedom (*e.g.*, electrons, phonons, excitons, and magnons) are intertwined, and their interplay is prominent to understanding the macroscopic properties of quantum materials.^1−3^ Photoexcitation with strong laser pulses provides a powerful method to drive correlated materials into out-of-equilibrium states and disentangle the dominant interactions (e.g., electron–electron correlation, spin–orbital coupling, and electron–phonon interaction). Upon photoexcitation, fundamental insights can be gained into light–matter interactions, photoinduced phase transitions, and ultrafast dynamics of quasi-particles.^4−14^

Photoinduced demagnetization of magnetic insulators paves the way for launching ultrafast dynamics of spins, which cannot be reached in terms of conventional methods to modulate the microscopic magnetism.^1,15,16^ Owing to the simple honeycomb crystal structure, the ruthenium-based compound α-RuCl_3_ provides an attractive platform to explore the physics of electronic correlations, unconventional magnetism, and optomagnetic effects in correlated insulators.^17−32^ It is illustrated that α-RuCl_3_ accommodates essential ingredients of the Kitaev model owing to the interplay of electron correlations and magnetic interactions, facilitating a variety of exotic quantum phases.^19−22^

Previous studies have found that α-RuCl_3_ has a substantial spin–orbit coupling and low-temperature magnetic order, matching the predictions of being a proximate quantum-spin liquid.^23^ Recent experiments using angle-resolved photoemission spectroscopy reported a bandgap of ∼1.0 eV, establishing α-RuCl_3_ as a spin–orbit coupling assisted magnetic insulator.^20^ Numerous studies have explored the possibility of reaching a phase having gapless spin excitations under magnetic fields and suggested that adequate perturbations are capable of triggering phase transitions among various magnetic phases and correlated states.^25−30^ Kasahara et al. argued that magnetic fields could destroy the long-range magnetic order and generate a quantum-spin-liquid state or lead to the fractionalization of spins into itinerant Majorana Fermions.^24^ More relevantly, experiments demonstrated that light pulses can be utilized to tailor the magnetic free-energy landscape of α-RuCl_3_ and that photoexcitation suffices to induce a quasi-stationary transient spin-disordered phase.^29,30^ However, it is elusive whether optical modulations with strong laser pulses can suppress the long-range magnetic order and introduce spin dynamics, which is a promising route to understand correlated physics and calls for studies of the underlying mechanism of photoexcitation under extreme conditions.

In this article, we employ ab initio calculations within the framework of real-time time-dependent density functional theory (TDDFT)^34,35^ to investigate laser-driven spin dynamics of the two-dimensional magnet α-RuCl_3_. To understand its optical response, we undertake a comprehensive evaluation of the electronic and magnetic properties of α-RuCl_3_, and further simulate its dynamical response to laser pulses with different photon energies and intensities. Based on the recently developed ACBN0 functional,^36−39^ the spin dynamics of the correlated insulator as well as ultrafast melting of the bandgap is explored. We find that the photoinduced demagnetization significantly depends on the laser wavelength on account of the photoexcited band renormalization (*i.e.*, insulator-to-metal transitions) and carrier excitation. The delicate interplay of the photodoping effect and the insulator-to-metal transition suggests a way to drive the electronic and magnetic structures out of equilibrium on a time scale of tens of femtoseconds.

## Electronic and Magnetic Properties of α-RuCl_3_ in the Ground State

**Figure 1:**
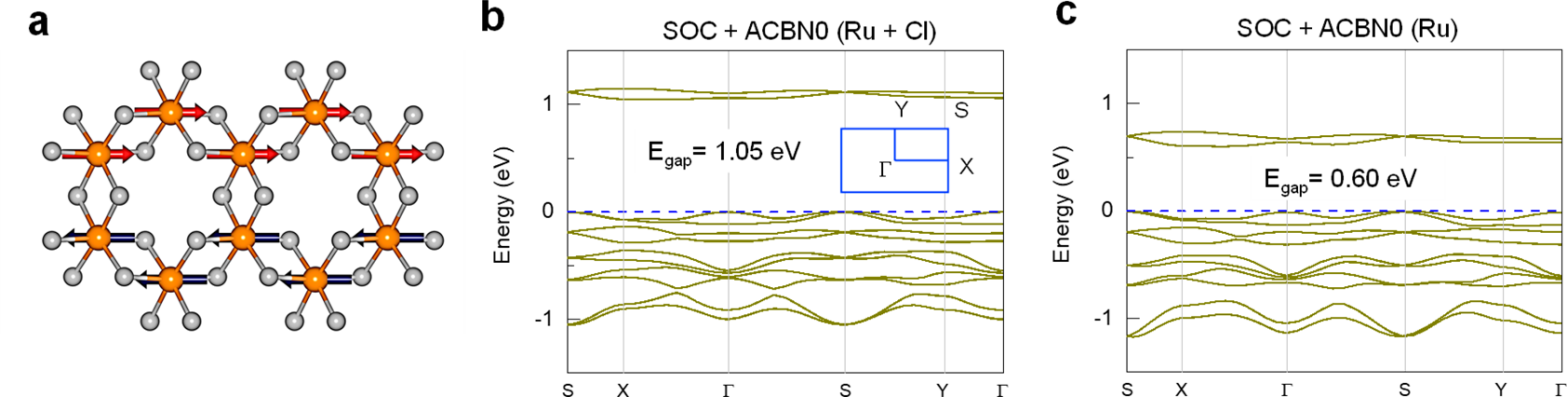
Atomic, magnetic, and electronic structures of α-RuCl_**3**_. (a) Atomic structure of α-RuCl_3_. Orange (gray) spheres denote Ru (Cl) atoms. Red and blue vectors indicate the magnetic moments in the ground state. (b) Band structure of α-RuCl_3_ with on-site Hubbard *U* correction on Ru and Cl orbitals. The effective Hubbard *U* values are respectively 1.96 and 5.31 eV for Ru 4d and Cl 3p orbitals after full self-consistency based on the ACBN0 functional. Inset shows a schematic of the Brillouin zone with high-symmetry points marked. (c) Band structure of α-RuCl_3_ with on-site Hubbard *U* on Ru 4d orbitals. The effective Hubbard *U* (*U*_*eff*_ = *U* – *J*) is 1.78 eV after full self-consistency. The dashed blue lines (0 eV) indicate the valence band maximum of each panel. Spin–orbital coupling (SOC) is included in all calculations.

Figure 1 exhibits the atomic, electronic, and magnetic structures of α-RuCl_3_. α-RuCl_3_ is a two-dimensional system with an ideal Ru honeycomb lattice, with the Ru–Cl–Ru angle being close to 90°. It hosts comparatively modest spin–orbit coupling in the 4d Ru ions of ∼0.1 eV.^19^ The ground state of α-RuCl_3_ displays an in-plane zigzag antiferromagnetic (AFM) order, in which the magnetic moments of Ru ions are parallel to other moments in the same zigzag chain and antiparallel to those in neighboring zigzag chains (Figure 1a).

We employ the recently proposed ACBN0 functional to exploit the electronic properties of the strongly correlated material.^36−39^ The functional is regarded as a pseudohybrid reformulation of the density-functional theory plus Hubbard *U* (DFT + *U*) method, enabling us to compute the Hubbard *U* and Hund’s *J ab initio* and self-consistently by solving generalized Kohn–Sham equations (Note 1 in Supporting Information). The method has been recently extended to the real-time case, within the framework of time-dependent density-functional theory. In practice, the functional is an efficient and computationally affordable method to study the optical response of correlated systems driven out of equilibrium.^36−39^

As to α-RuCl_3_, the converged effective Hubbard *U* terms (*U*_*eff*_ = *U* – *J*) with the ACBN0 functional are 1.96 eV for Ru 4d orbitals and 5.31 eV for Cl 3p orbitals, respectively. The parameters yield an indirect bandgap of E_gap_ = 1.05 eV (Figure 1b), in excellent agreement with the experimental observation (∼1.0 eV).^20^ In contrast, the indirect bandgap of α-RuCl_3_ with on-site Hubbard *U* on only Ru 4d orbitals reduces to 0.60 eV (Figure 1c), in accordance with previous calculations using empirical Hubbard *U* terms.^31,32^ Furthermore, without on-site Hubbard corrections, the band structure of α-RuCl_3_ displays a metallic state. These results validate the necessity of on-site terms on both Cl 3p and Ru 4d orbitals.

In previous studies, theoretical calculations based on density functional theory plus a Hubbard correction yield a relatively small bandgap (0.6–0.8 eV) in α-RuCl_3_^31,32^ while recent experiments reported a larger bandgap of ∼1.0 eV,^20^ indicating an evident inconsistency. With respect to magnetic moments, Banerjee et al.^19^ reported the ordered moments in Ru^3+^ ions are around 0.4 μ_B_ for the low-temperature magnetic phase using neutron diffractions. Based on the ACBN0 functional, the magnetic moments of the Ru atoms are 0.33 μ_B_ for the in-plane zigzag AFM order (with Hubbard correction on both orbitals), which is considered to be the ground-state magnetic configuration. These findings reflect that those calculations with both spin–orbit coupling and Hubbard *U* corrections on the Ru 4d and Cl 3p orbitals are able to capture the microscopic interactions and reproduce the experimental electronic structures.

From the projected band structures of α-RuCl_3_ (Figure S1), the orbitals of both the conduction and valence bands are found to exhibit non-negligible contributions from Cl orbitals, validating that on-site Coulomb potentials on both the Ru 4d and Cl 3p orbitals are critical to obtain accurate electronic and magnetic properties. This is interpreted as the on-site Coulomb potential on the chlorine ions increasing the localization of the lone pairs and, hence, the bandgap. Besides, we calculated the amount of charge transfer in the compounds. From Bader analysis (Note 1), the Ru atoms in α-RuCl_3_ have the charge of 6.94 *e*, in contrast to 8 *e* in the pristine valence orbitals of the pseudopotential. On the other hand, the charge of each Cl atom is 7.35 *e* (out of 7 *e*), denoting a significant charge transfer. Therefore, the orbitals of Ru atoms cannot be considered fully localized, and the use of large Hubbard *U* as a fitting parameter lacks a reasonable physical basis.

Besides the in-plane AFM state, another possible modulated zigzag antiferromagnet order is observed where the magnetic moments are oriented ±35° from the ab plane in experiments.^21^ Our further simulations of α-RuCl_3_ with modulated magnetic orders are presented in Figure S2, where the magnetic orders are fixed as the starting parameters. For the two magnetic states, the energy difference is very small (10 meV/atom energetically lower for the modulated zigzag state). We observe that α-RuCl_3_ with the modulated zigzag state exhibits a slightly smaller bandgap of 1.0 eV while the in-plane ferromagnetic state shows a much smaller indirect bandgap of 0.80 eV. The comparison indicates magnetic states are crucial to determine the electronic structures of the system. It should be mentioned that our calculations do not consider the interlayer magnetic interactions because it is hard to resolve the interlayer structure of α-RuCl_3_ in recent experiments.^19^ The weak van der Waals bonding between the α-RuCl_3_ layers enables several stacking configurations, including a rhombohedral phase with space group R3, as well as a *C*2/*m* phase. In this regard, the monolayer α-RuCl_3_ is used as a prototype to investigate the magnetic structure and photoinduced response, as done in other studies.^29,30^

## Wavelength Dependence of Spin Dynamics

**Figure 2:**
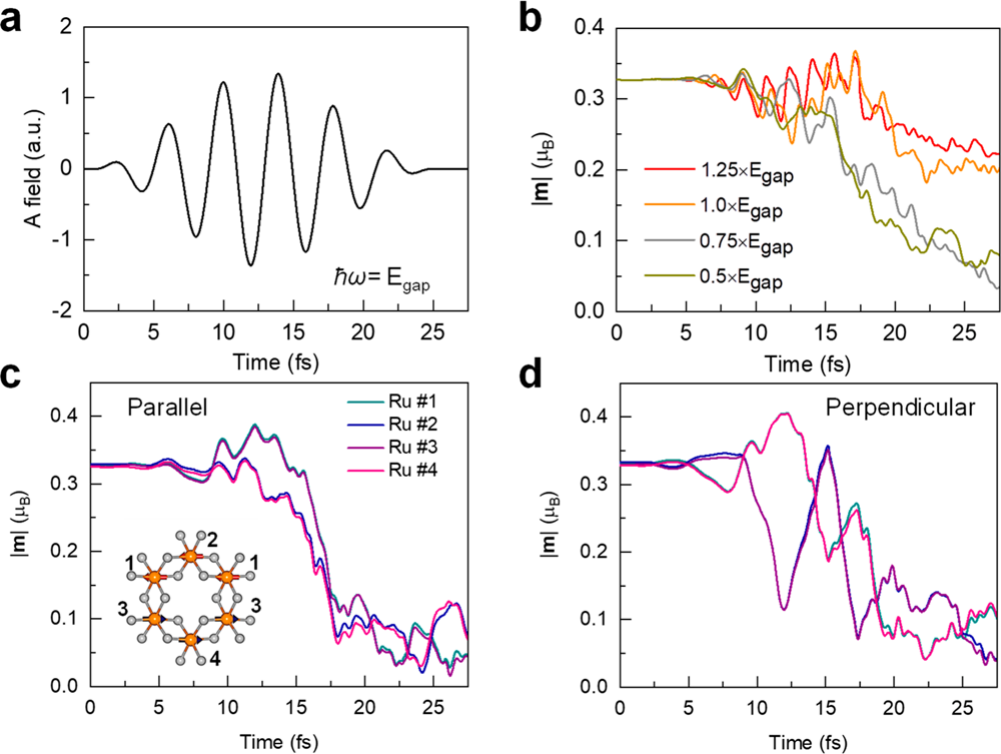
Laser-induced spin dynamics in α-RuCl_3_. (a) Time-dependent vector potential for a wavelength of 1180 nm. Applied electric fields are in-plane and polarized perpendicularly to the magnetic moment of Ru atoms of α-RuCl_3_ with photon energies above or below the ground-state bandgap. The peak value of the laser is at 12.7 fs. (b) Time evolution of magnetic moments of Ru atoms under laser excitations with different photon energies (*ℏω=*1.25 ×, 1.0 ×, 0.75 ×, and 0.5 × E_gap_, respectively). In panels (a) and (b), the laser intensities correspond to I_0_ = 2.5 × 10^12^ W/cm^2^ for the different photon energies. |**m**| indicates the averaged magnet over the values of four Ru atoms in the supercell. (c) Dynamics of the magnetic moment of four Ru atoms for laser pulses with parallel polarization and *ℏω=* 0.5 × E_gap_. For in-plane polarization parallel to the Ru moments, Ru #1 follows the dynamics Ru #3, and #2 goes with #4. Inset shows the labels of the Ru atoms. (d) The same quantities as shown in (c) for the laser pulses with the polarization perpendicular to the Ru moments. In the perpendicular case, we find that Ru #1 follows the same trend with Ru #4 and Ru #2 with Ru #3. The magnetic moment is calculated by averaging over a sphere of radius 2.22 Bohr around the Ru atoms.

In the following, we focus on the photoinduced out-of-equilibrium dynamics in α-RuCl_3_ by altering the laser photon energies. The typical shape of the electric field introduced by the applied laser pulse is shown in Figure 2a, with a wavelength of 1180 nm, whose photon energy corresponds to the bandgap (1.05 eV) of α-RuCl_3_. Figure 2b summarizes the real-time evolution of the magnetic moments (|**m**|, averaged over the four Ru atoms in the supercell) in α-RuCl_3_ under different photon energies above and below the bandgap. The pump laser intensity (2.5 × 10^12^ W/cm^2^) in Figure 2a corresponds to 6.35 × 10^–2^ mJ/cm^2^, which is on the same order with recent experiments.^33^

For a photon energy at the bandgap, a closer inspection of the magnetic moments reveals a clear drop in 25 fs, after which they become relatively stable with only a small fluctuation. For lower photon energies, ultrafast melting of the zigzag AFM magnetic order is observed on a time scale of 20 fs. For a photon energy higher than the bandgap (ℏω = 1.25 × E_gap_), the averaged magnetic moments reduce to 0.23 μ_B_ and oscillate slightly afterward. We find faster demagnetization processes when considering longer wavelengths corresponding to lower photon energies (Figure 2b). Notably, the residual magnetic moment for *ℏω =* 0.75 × E_gap_ is roughly 0.04 μ_B_ at the end of laser irradiation and similar to the value for ℏω = 0.5 × E_gap_, reflecting saturation of the ultrafast demagnetization (see Figure S3 for snapshots of magnetic moments of α-RuCl_3_ under laser excitation). The wavelength dependency of spin dynamics provides a novel knob for the high modulation of magnetic states under laser excitation and deserves elaborate investigations.

Given the equilibrium results in Figure. 1, it is clear that the light-induced reduction of *U* is crucial for the observed changes in the magnetic structure. In addition, we observe similar demagnetization for a longer laser pulse of 50 fs, as shown in Figure S4. To understand the above findings, we monitored the effective Hubbard *U* of the Ru and Cl orbitals. It is noteworthy that the laser decreases the effective *U* for the Ru 4d orbital to 1.50 eV for ℏω = 0.5 × E_gap_ (Figure S5). The modification is obviously faster with regard to ℏω =1.25 × E_gap_, in which the residual effective *U* is 1.39 eV. Since the optical excitation is an ingredient of paramount importance to tune the magnetic properties of correlated materials (*e.g*., charge-transfer insulators^38^ and Weyl semimetals^39^), dynamical modification of electron–electron correlations may pave the way to investigate the phase transitions in α-RuCl_3_ from a new degree of freedom.

The variations of the magnetic moments of individual Ru atoms are also provided in Figure 2c,d. For the perpendicular polarization, the photoexcited dynamics demonstrate that the magnitude of spins on different sublattices oscillates with significant out-of-phase components (panel (d) and Figure S3) and the change follows the frequency of the applied laser pulse. It could be attributed to laser-induced symmetry breaking in charge distributions. We should note that laser pulses with different polarizations are capable of breaking the different symmetries of α-RuCl_3_, bringing about different spin sublattices in the dynamics. For the perpendicular polarization, laser excitation breaks the mirror plane vertical to the magnetic moments, and we observe two distinct sublattices for demagnetization of Ru orbitals. This is attributed to the symmetry-breaking and the charge redistribution induced by perpendicular excitations (Figure S6).

## Intensity and Polarization Dependence of Spin Dynamics

**Figure 3:**
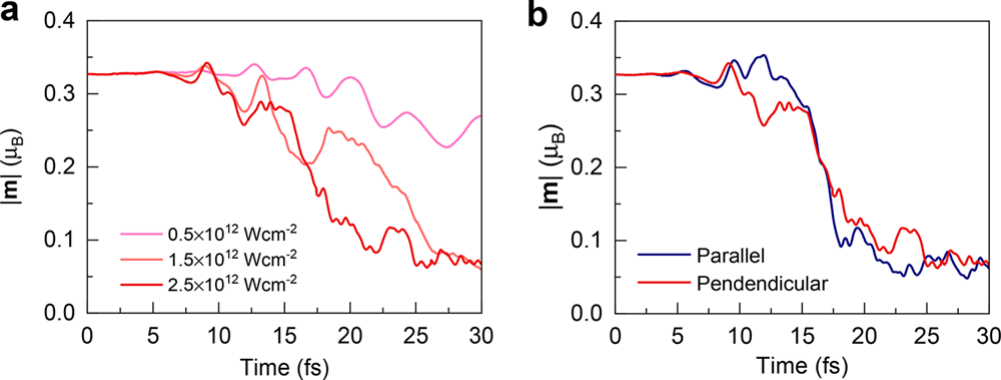
Intensity and polarization dependence of the light-induced spin dynamics in α-RuCl_3_. (a) The intensities correspond to I_0_ = 0.5 ×, 1.5 ×, and 2.5 × 10^12^ W/cm^2^, respectively. The laser pulse with perpendicular polarization and higher intensity introduces larger modulation of the atomic magnetic moments. We take the photon energy of ℏω = 0.5 × E_gap_ as an example. (b) The influence of laser polarization on spin propagation. Parallel (perpendicular) direction denotes the laser polarization along (perpendicular) to the magnetic moments shown in Figure 1a. The driving intensity and photon energy of laser pulses are I_0_ = 2.5 × 10^12^ W/cm^2^ and ℏω = 0.5 × E_gap_, respectively.

We also investigated the impact of laser intensity on ultrafast demagnetization. From Figure 3a, it is clear that the laser pulse with a stronger intensity introduces a more considerable modulation of the atomic magnetic moments. For an intensity of I_0_ = 0.5 × 10^12^ W/cm^2^, the residual magnetic moment is 0.21 μ_B_, and the zigzag AFM state is still stable after the laser illumination. Whereas for I_0_ = 2.5 × 10^12^ W/cm^2^, we obtain a saturation of the demagnetization at 0.06 μ_B_, indicating a stronger reduction and complete melting of the magnetic structure.

Figure 3b illustrates the laser-induced spin dynamics for the laser pulses with the polarization parallel to the spins. The spin dynamics follow trends similar to those with perpendicular polarization and introduce a distorted state with a residual magnetic moment of 0.04 μ_B_, confirming ultrafast demagnetization is robust for the parallel polarization. Our further analysis demonstrates that dynamical modification of the electronic and magnetic parameters in strongly correlated magnets is indeed possible by purely optical means without involving the crystal lattice dynamics. It should be noted that the gap is sensitive to both Hubbard terms and the magnetic orders, indicating that α-RuCl_3_ is a Mott–Slater insulator. Laser polarization may also be a cardinal ingredient of importance to control the magnetic structures.

## Photoinduced Insulator-to-Metal Transition in α-RuCl_3_

**Figure 4:**
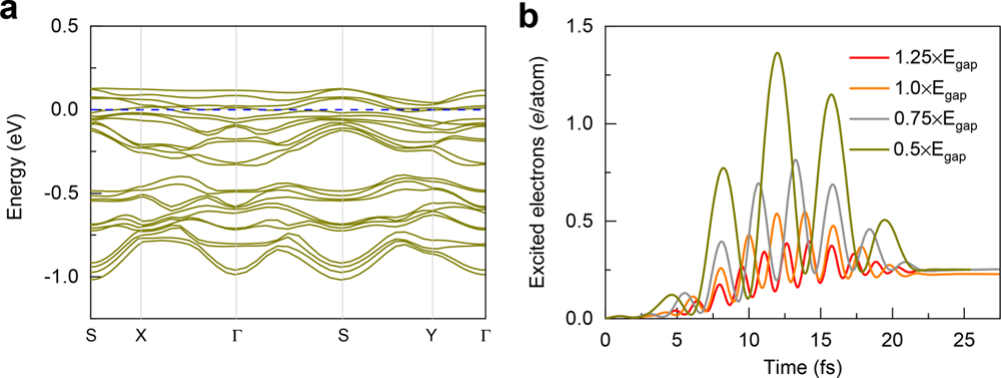
Photoinduced insulator-to-metal transition and carrier populations for various photon energies. (a) Time-resolved band structures for the laser with the photon energy of ℏω = 0.5 × E_gap_ for α-RuCl_3_ at 25 fs. The dashed blue line represents the Fermi level of the system. (b) The photon energies range from ℏω = 0.5 × to 1.25 × E_gap_. The number of excited electrons is calculated based on the projections of the time-evolved wave functions on the ground-state wave functions. Here, the intensity is I_0_ = 2.5 × 10^12^ W/cm^2^ and the polarization is perpendicular to the magnetic moments. The results indicate that smaller photon energies lead to higher excited carrier densities owing to the collapse of the bandgap.

We carried out comprehensive calculations for the out-of-equilibrium electronic properties and photoinduced carrier excitations in α-RuCl_3_. Figure 4a exhibits the transient band dispersion of photoexcited α-RuCl_3_ for the photon energy of ℏω = 0.5 × E_gap_ (see Figure S7 for the full trajectory and corresponding bandgaps). The transient band structures and bandgaps are computed from the time-evolved density under various laser excitations; see Note 1 in Supporting Information. The bandgap drops strikingly to 0.24 eV before the spin subsystem responds significantly (in 10 fs). After that, the bandgap melts completely when the laser pulse reaches the peak at about 15 fs, revealing that the band renormalization can take place without any structural distortions in α-RuCl_3_. We interpret the ultrafast collapse of the bandgap or insulator-to-metal transition in several tens of femtoseconds as indicating that the strong laser pulses greatly change the electron correlations (illustrated by the effective Hubbard terms) and lead to the modification of magnetic structures.

Furthermore, the ultrafast insulator-to-metal transition is robust for various photon energies at the laser with a strong intensity (Figure S8). Regarding all photon energies, the ultrafast band renormalization takes place within 15 fs. This explains the smaller modulation of magnetic moments. Therefore, the bandgap of α-RuCl_3_ can be easily modulated by optical excitation. As a direct consequence, the required excitation energy for Zener tunneling or multiphoton ionization decreases during laser irradiation in α-RuCl_3_.

In order to elucidate the origin of the optical response, the excited carrier densities are analyzed by characterizing the charge excitation from the valence to conduction bands of Kohn–Sham orbitals, which are calculated by projecting the time-evolved wave functions on the ground-state wave functions of α-RuCl_3_ (see SI for more details), as displayed in Figure 4b. For ℏω = 1.25 × E_gap_, the photoinduced carrier population increases to 0.40 *e*/atom within 15 fs and then oscillates around 0.25 *e*/atom. Following the shapes of the laser pulses, the peak of the carrier density reaches 1.36 *e*/atom for ℏω = 0.5 × E_gap_. The excited carrier concentration is also sensitive to the photon energies of the laser; i.e., longer wavelengths result in more significant carrier densities. This is attributed to the laser-induced collapse of the bandgaps and the laser pulses with smaller photon energies becoming resonant with the transient bandgaps.

To validate the physical picture, we performed additional simulations from the modulated zigzag antiferromagnet order to track the magnetic dynamics and transient band structures (Figure S9). It is obvious that the laser-induced magnetic and electronic dynamics are similar for the two possible magnetic states (in-plane zigzag and modulated zigzag orders). Therefore, we obtain a robust picture of an ultrafast photoinduced insulator-to-metal transition in α-RuCl_3_. The collapse of the bandgap occurs when the electrons are excited by strong laser pulses. The saturation at the half of the ground-state bandgap is interpreted as the excited carrier density being high enough to modulate the electronic structures and transient bandgaps and introduce the rapid demagnetization. In addition, nonlinear excitation processes can also play a role in the wavelength dependence of the demagnetization, especially at the beginning of the strong laser pulses. Our findings support that the photoinduced carriers are important to introduce ultrafast melting of magnetic structures. Notably, the recovery process after demagnetization is not traced in this work because the real-time TDDFT method incorporates no effective energy dissipation channel.

The direct electronic and spin dynamics obtained from our first-principles calculations enable us to simulate the photoinduced response of α-RuCl_3_ at the atomistic spatial scale on a femtosecond time scale. Strong photoexcitation leads to an ultrafast insulator-to-metal transition and creates a high density of electron–hole pairs. The magnetic interactions are modulated as an effective nonmagnetic state is obtained. It should be noted that the ultrafast process is not the result of collective magnetic excitation (e.g., magnons), which would keep the magnitude of the magnetic moment fixed while decreasing its components along the local order parameter directions. We note that extracting information about the collective excitation should demand more effort from the model Hamiltonian and TDDFT methods.^34,35^

For α-RuCl_3_, laser excitations significantly modify the Hubbard terms and generate a substantial number of electron–hole pairs, triggering a rapid insulator-to-metal transition. In the meantime, excess energy in the electron system transfers to the spin subsystem, inducing ultrafast demagnetization. The interplay between the photodoping effect and the insulator-to-metal transition plays a vital role in the magnetic dynamics, while the collapse of magnetic structures causes the reduced bandgap in return. Notably, we cannot simply view the demagnetization as only a consequence because the reduced magnetic moments contribute to the decreased bandgaps.^25^ Upon significant modification of the Hubbard terms, the local magnetic moments and the Mott gap are found to vanish simultaneously. Our results indicate that both the magnetic ordering and the Hubbard terms are crucial to stabilize the insulating state.

Regarding the laser energies exceeding the ground-state bandgap, photoexcitation creates a dense population of electron–hole pairs. This, in turn, triggers the closure of the bandgap and initiates an ultrafast demanganization process. In contrast, when laser energies below the ground-state bandgap are employed, a concentration of electron–hole pairs is generated as well, coupled with the presence of in-gap states and orbital excitations. Consequently, the excited carriers play a substantial role in modifying the electronic structures, eventually inducing an intriguing insulator-to-metal transition. As the energy of the pump laser surpasses the transient bandgaps, the trends and time scales governing laser-induced magnetic dynamics exhibit comparable behavior and minimal discrepancies for both above- and below-gap excitations. In experiments, the magnetic transition could be probed by the time-resolved linear magnetic dichroism and X-ray magnetic circular dichroism,^31,32^ which is able to illustrate the magnetic difference. Photoinduced insulator-to-metal transition in α-RuCl_3_ can be detected by time- and angle-resolved photoemission spectroscopy. It is noteworthy that a femtosecond laser-induced quantum-spin-liquid state is beyond the current study and will be the subject of future studies.

In conclusion, our *ab initio* simulations revealed the nature of photon-driven electron and spin dynamics in α-RuCl_3_. We demonstrated that laser pulses can provoke a magnetic transformation between zigzag AFM magnetic order and disordered magnetic states with much smaller magnetic moments. In addition, the spin response is remarkably sensitive to the laser wavelength and polarization, on account of the photoinduced insulator-to-metal transition and different excited carrier distributions. This subtle interplay suggests a way to modulate the electronic and magnetic structures by using ultrashort laser pulses. Our work provides new insights into photoexcitation-induced magnetic phase transitions and may pave the way for suppressing the long-range magnetic order and realizing a quantum-spin-liquid state at ultrashort time scales.

## Supporting Information Available

Detailed methods, parameters, and additional figures (PDF)
