# Supplementary material for: Ultrafast Spin Dynamics and Photoinduced Insulator-to-Metal Transition in α-RuCl3
Source: Nano Lett. 2023 Sep 11;23(18):8712–8. doi: 10.1021/acs.nanolett.3c02668 (PMC10540253; doi:10.1021/acs.nanolett.3c02668)
Supplement: Supplementary file 1 — nl3c02668_si_001.pdf [file nl3c02668_si_001.pdf]

Supporting Information for

Ultrafast Spin Dynamics and Photoinduced Insulator-to-Metal Transition in α-$RuCl_3$

Jin Zhang[1,*], Nicolas Tancogne-Dejean[1], Lede Xian[1,7], Emil Viñas Boström[1], Martin Claassen[3], Dante M. Kennes[1,4], and Angel Rubio[1,2,5,6*]

[1]*Max Planck Institute for the Structure and Dynamics of Matter and Center for Free-Electron Laser Science, Luruper Chaussee 149, 22761, Hamburg, Germany*

[2]*Center for Computational Quantum Physics (CCQ), The Flatiron Institute, 162 Fifth avenue, New York NY 10010*

[3]*Department of Physics and Astronomy, University of Pennsylvania, Philadelphia, PA 19104, USA*

[4]*Institut für Theorie der Statistischen Physik, RWTH Aachen University and JARA-Fundamentals of Future Information Technology, 52056 Aachen, Germany*

[5]*Nano-Bio Spectroscopy Group, Universidad del País Vasco, 20018 San Sebastián, Spain*

[6]*Center for Computational Quantum Physics (CCQ), The Flatiron Institute, 162 Fifth Avenue, New York NY 10010*

[7]*Songshan Lake Materials Laboratory, Dongguan 523808, China*

**Corresponding: Angel Rubio (angel.rubio@mpsd.mpg.de)*

Jin Zhang (jin.zhang@mpsd.mpg.de)

This file contains:

Note 1. Methods and parameters

S1. Projected band structures of α-$RuCl_3$

S2. Band structure of α-$RuCl_3$ with different magnetic orders.

S3. Snapshots of magnetic moments of α-RuCl3 under laser excitation at different photon energies

S4. Magnetic moment dynamics for a laser with a duration of 50 fs.

S5. Laser-driven dynamics of effective Hubbard *U* of Ru and Cl orbitals

S6. Charge redistribution for α-$RuCl_3$ in laser induced dynamics

S7. Dynamics of the electronic band structure induced by laser excitation

S8. Time-dependent band structures under laser excitation

S9. Laser-induced spin dynamics staring from a modulated zigzag magnetic order in α-$RuCl_3$

Note1. Methods and parameters

TDDFT simulations. The evolution of the spinor states and the evaluation of the time-dependent Hubbard *U* and magnetization are computed by propagating the generalized Kohn–Sham equations within time-dependent density functional theory including mean-field interactions, as provided by the Octopus package [1-2], using the ACBN0 functional together with the local density approximation (LDA) functional for describing the semi-local DFT part. We compute *ab initio* the Hubbard *U* and Hund's *J* for the 4d orbitals of Ruthenium and 3p orbitals of Chlorine. In the time-dependent simulations, the laser is coupled to the electronic degrees of freedom via the standard minimal coupling prescription using a time-dependent, spatially-homogeneous vector potential A(t), with the electric field $\mathrm{E(t)} = -\frac{1}{c}\frac{\partial A(t)}{\partial t}$, where c is the velocity of light in vacuum. We consider a laser pulse of 12.7 fs duration at full-width half maximum with a sin-square envelope corresponding to a total width of 25.4 fs. In all our calculations, a carrier-envelope phase of $\phi = 0$ is used.

The experimental lattice parameters (*i.e.*, 5.98 Å and 10.35 Å) and atomic positions are employed [3]. We employed a mixed periodic boundary condition with a vacuum region of 15 Å to ensure that interactions between periodic images were negligible. We employ norm-conserving HGH pseudopotentials, a real-space grid spacing of 0.33 atomic units, and an 8 × 6 × 1 *k*-point grid in a 1×√3 supercell with a rhombus shape (containing 16 atoms for the zigzag AFM magnetic order). The inclusion of semi-core states of Ru and Cl elements are prominent to obtain accurate electronic structures; the valence electrons explicitly included are Ru: $4s^2$, $4p^6$, $4d^7$ and $5s^1$; Cl: $3s^2$ and $3p^5$. In all calculations, we include the spin-orbit coupling, which is vital to obtain the correct electronic and magnetic structures. In this work, we neglect the coupling to phonons and the contribution from thermal effects to the demagnetization.

ACBN0 functional implementation. The time-dependent generalized Kohn-Sham equation (in atomic units) within the adiabatic approximation is simply expressed by

$$i\frac{\partial|\psi_i^\sigma(t)\rangle}{\partial t} = \left[\frac{-\nabla^2}{2} + v_{ext}(t) + v_H[n(\boldsymbol{r},t)] + v_{xc}[n(\boldsymbol{r},t)] + V_U[n(\boldsymbol{r},t),\{n_{mm'}^\sigma\}]\right]|\psi_i^\sigma(t)\rangle. \quad (1)$$

where $\psi_{n,k}^\sigma(t)$ is a Bloch state with a band index n, at the point k in the Brillouin zone and with the spin index σ, v_{ext} is the external potential containing both the driving laser field and the ionic potential, v_H is the Hartree potential, v_{xc} is the exchange-

correlation potential, and V_U is the plus U (nonlocal) operator.

$$V_U[n(\boldsymbol{r},t),\{n^{\sigma}_{mm'}\}] = U_{eff}\sum_{m,m'}\left(\frac{1}{2}\delta_{mm'} - n^{\sigma}_{mm'}\right)P^{\sigma}_{m,m'}. \quad (2)$$

Here $P^{\sigma}_{m,m'} = |\delta^{\sigma}_{m}\rangle\langle\delta^{\sigma}_{m'}|$ is the projector over the localized subspace defined by the localized orbitals $\{\phi^{\sigma}_{m}\}$, and n^{σ} is the density matrix of the localized subspace.The number of excited electrons (N_{ex}) is calculated by projecting the time-evolved wavefunctions ($|\psi_{n,k}(t)\rangle$) on the basis of the ground-state wavefunctions ($|\psi^{GS}_{n',k}\rangle$).

$$\boldsymbol{N_{ex}(t) = N_e - \frac{1}{N_k}\sum^{occ}_{n,n'}\sum^{BZ}_{k}\left|\langle\psi_{n,k}(t)|\psi^{GS}_{n',k}\rangle\right|^2}. \quad (3)$$

where N_e is the total number of electrons and N_k is number of k-points used to sample the Brillouin zone (BZ). The sum over band indices n and n' go over all occupied states.

This method has been recently extended to the real-time case, within the framework of time-dependent density-functional theory [4-5]. In practice, the ACBN0 functional is an efficient and computationally affordable method to simulate the optical response of correlated systems driven out of equilibrium without relying on perturbation theory. The method has been proven to yield accurate electronic bandgaps, effective *U*, and other electronic properties of the charge-transfer insulators and magnetic insulators in pyrochlore iridates [6-7].

In our simulations, the SOC comes from the pseudopotentials and is therefore fixed during the entire simulation. Concerning the magnetic interactions and correlations, they depend directly on the Hubbard U terms, which change in the simulations. The magnetic interactions go as t^2/U, where t is the kinetic energy. The kinetic energy of the electrons will increase with the laser pulse, and hence t will increase. Hubbard U is found to decrease in the meantime. So, we expect the values of parameters increase during the laser excitation.

Bader charge analysis. The calculations were performed within the Vienna Ab initio Simulation Package (VASP) [8-9] using a projector-augmented wave (PAW) pseudopotential in conjunction with the Perdue–Burke–Ernzerhof (PBE) functionals and plane-wave basis set with energy cutoff at 400 eV [10]. For Bader analysis [11], the surface Brillouin zone was sampled by 8×6×1 Monkhorst–Pack k-meshes. All structures were fully relaxed until the force on each atom was less than 0.01 eV $Å^{-1}$. We checked and reproduced the band structures with Hubbard correction on both Ru and Cl orbitals in α-$RuCl_3$, yielding a quantitatively consistent Mott gap of 1.02 eV using VASP.

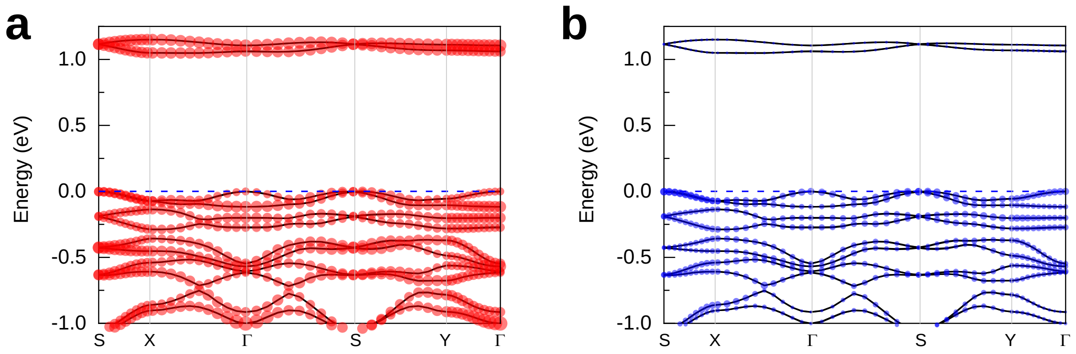

Figure S1. Projected band structures of α-$RuCl_3$. Projected band structures of α-$RuCl_3$, with ACBN0 functional on both Ru (red dots on the left) and Cl elements (blue dots on right). The dashed blue lines (0 eV) indicate the valence band maximum of each panel.

From Figure S1, we find that the states at the top of the valence results from a hybridization of Ru and Cl orbitals. The states at both the conduction bands and valence bands exhibit nonnegligible Cl-character, validating that both the on-site Coulomb potentials on Ru 4d and Cl 3p orbitals are crucial to obtain accurate electronic properties.

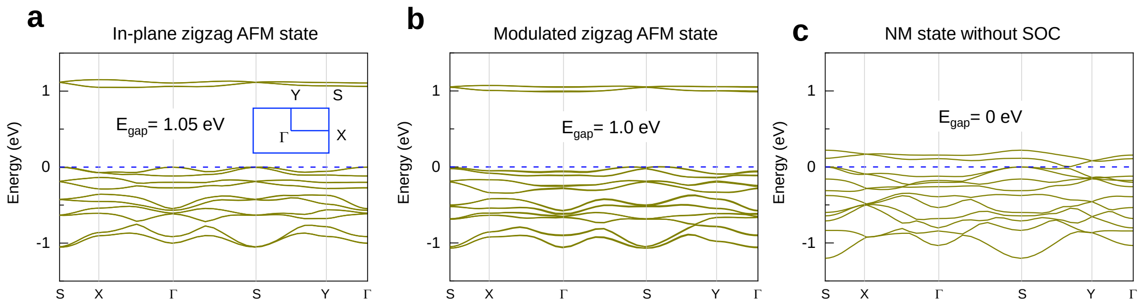

Figure S2. Band structure of α-RuCl$_3$ with different magnetic orders. (a) Band dispersions for in-plane zigzag antiferromagnet order. (b) Band dispersions for modulated zigzag antiferromagnet order where the magnetic moments are oriented ±35° from the ab plane. In panel a-b, spin-orbital coupling (SOC) and Hubbard corrections for both the Ru and Cl orbitals are included. (c) Electronic structures of RuCl$_3$ without SOC and non-magnetic state. The effective Hubbard U values are respectively 2.16 eV and 5.42 eV for Ru 4d and Cl 3p orbitals after full self-consistency based on ACBN0 functional. The dashed blue lines (0 eV) indicate the valence band maximum of each panel.

Our simulations on α-RuCl$_3$ with the modulated out-of-plane magnetic orders are presented in Figure 2, where the magnetic orders are artificially fixed as the starting parameters. We observe that α-RuCl$_3$ with the modulated zigzag state exhibits a slightly smaller bandgap of 1.0 eV. The comparison indicates magnetic states are crucial to determine the electronic structures of the system. If the SOC is neglected, the band structure is significantly affected. Moreover, without SOC, no demagnetization would occur.

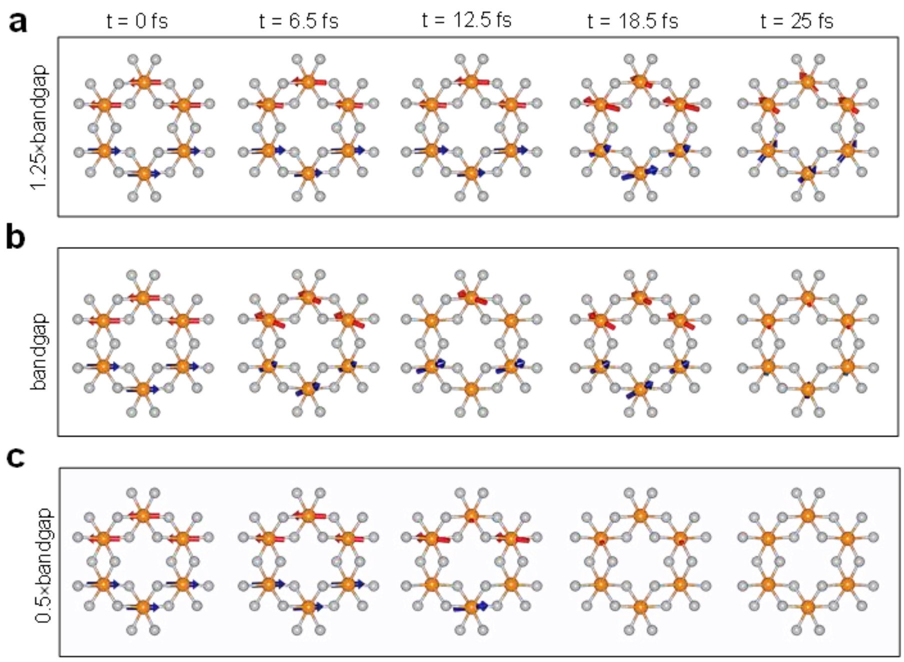

Figure S3. Snapshots of magnetic moments of α-$RuCl_3$ under laser excitation at different photon energies. (**a**) Time-dependent magnetic moments for the laser pulse with the photon energy of $\hbar\omega$=1.25×bandgap at 0, 6.5, 12.5, 18.5 and 25 fs, respectively. (**b**) The same quantities as (a) for the photon energy corresponding to the ground-state bandgap of α-$RuCl_3$. (**c**) The same quantities as (a) for the photon energy corresponding to half of the bandgap. The laser intensity is $I_0=2.5\times10^{12}$ W/cm^2 and the polarization is perpendicular to the magnetic moments. For clarity, the snapshots for $\hbar\omega$=0.75×bandgap are not shown here considering they are quite similar to those for $\hbar\omega$=0.5×bandgap.

To have a clear view, Figure S3 shows snapshots of magnetic moments at different times during laser illumination. As illustrated in Figure 3a, the residual moments for ħω=1.25×bandgap are still non-negligible at 25 fs, leading to a disordered magnetic structure with apparent rotations. In contrast, the magnetic moments reduce to a negligible value in 20 fs for ħω=0.5×bandgap. We should note that laser pulses with different polarizations can break different mirror planes, bringing about different spin sublattices for the dynamics. For the perpendicular polarization, laser excitation breaks the mirror plane vertical to the magnetic moments and we observe two distinct sublattices.

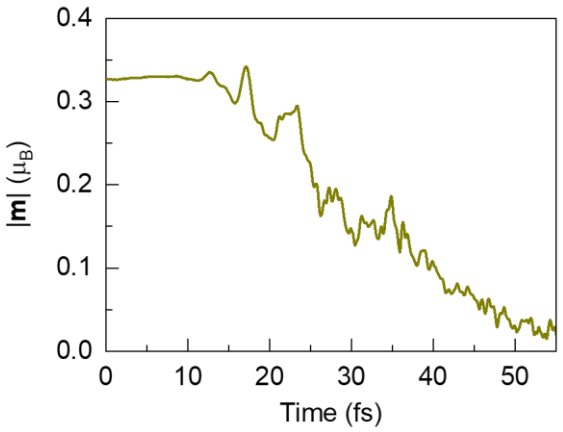

Figure S4. Magnetic moment dynamics for a laser with a duration of 50 fs. The photon energy corresponds to the ħω=0.5×Egap. The laser intensity is $I_0=2.5\times10^{12}$ W/cm^2 and the polarization is perpendicular to the magnetic moments.

For the simulation, we observe similar demagnetization for the laser pulse with the durations of 25 fs and 50 fs.

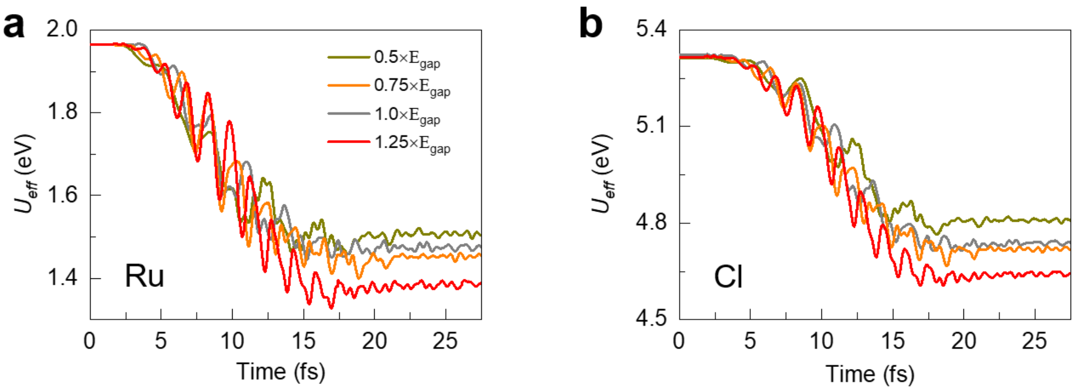

Figure S5. Laser-driven dynamics of effective Hubbard *U* of Ru and Cl orbitals. (**a**) Dynamics of U_{eff} for the Ru 3d orbitals for the photon energies displayed in Figure 2b. (**b**) The same quantities as shown in (a) for the Cl 2p orbitals. The laser pulses are in perpendicular polarization.

In Figure S5, we monitored the effective Hubbard U of Ru and Cl orbitals. It is obvious that the laser decreases the effective U for Ru 4d orbital to 1.50 eV for $\hbar\omega=0.5\times E_{gap}$. The modification is obviously faster with regard to $\hbar\omega=1.25\times E_{gap}$, in which the residual effective U is 1.39 eV.

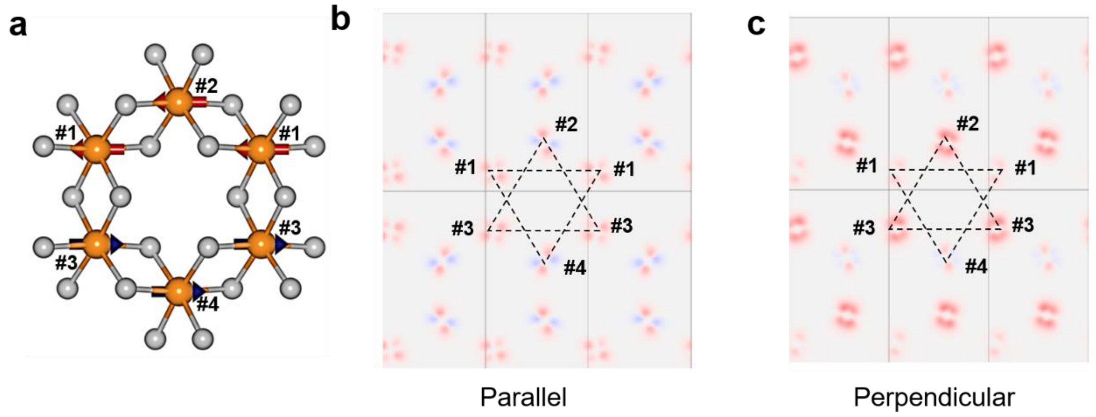

Figure S6. Charge redistribution for α-$RuCl_3$ in lase-induced dynamics. (**a**) Atomic structure and corresponding orders of the 4 atoms in primitive cell of α-$RuCl_3$. (**b**) The spatial charge distribution (in-plane cut) after photoexcitation with a parallel laser pulse. (**c**) The spatial charge distribution after photoexcitation with a perpendicular laser pulse. The photon energy corresponds to the $\hbar\omega$=0.5×E_{gap} and the laser intensity is I_0=2.5×10^{12} W/cm^2.

From Figure S6, the laser pulses with different polarizations are capable of breaking the different symmetries of α-$RuCl_3$, bringing about different spin sublattices in the dynamics. For the perpendicular polarization, laser excitation breaks the mirror plane vertical to the magnetic moments and we observe two distinct sublattices for demagnetization of Ru orbitals.

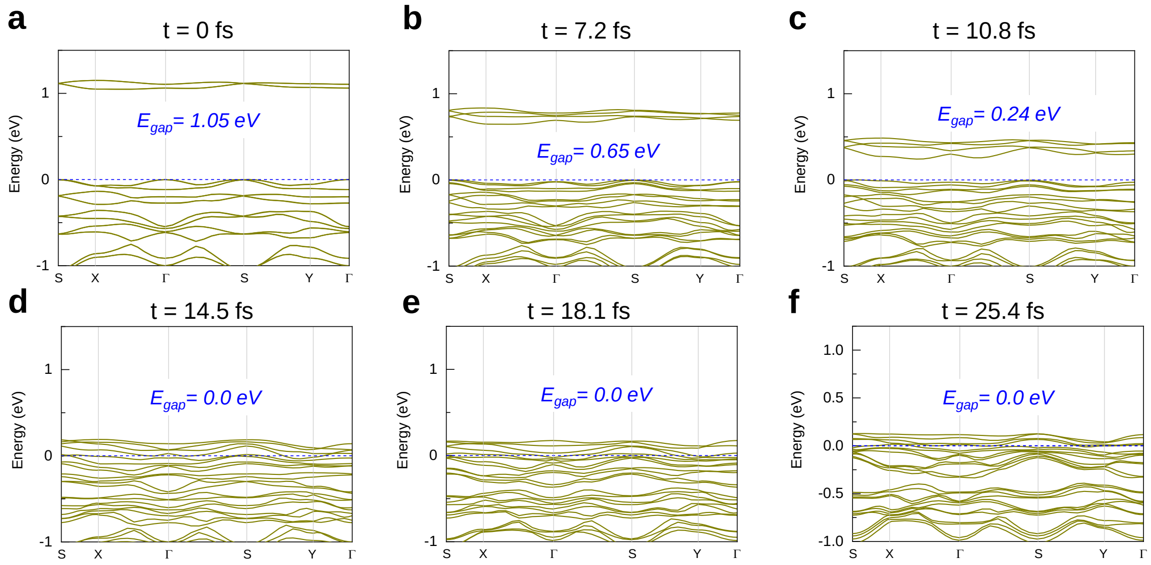

Figure S7. Time-dependent band structures under laser excitation. Time-dependent band structures for the laser with the photon energy of $\hbar\omega$=1.25× E_{gap} of α-$RuCl_3$ at 0, 7.2, 10.8, 14.5, 18.1 and 25.4 fs after photoexcitation, respectively. We use $I_0=2.5\times10^{12}$ W/cm^2 and the polarization is perpendicular to the magnetic moments as an example. As time goes, the value of Mott gap melts completely in 15 fs.

Figure S7 exhibits the full trajectory and corresponding bandgaps. We find the bandgap drops strikingly to 0.24 eV before the spin subsystem responds significantly (in 10 fs). After that, the bandgap melts completely when the laser pulse reaches the peak at about 15 fs, revealing that the band renormalization can take place without any structural distortions in α-$RuCl_3$.

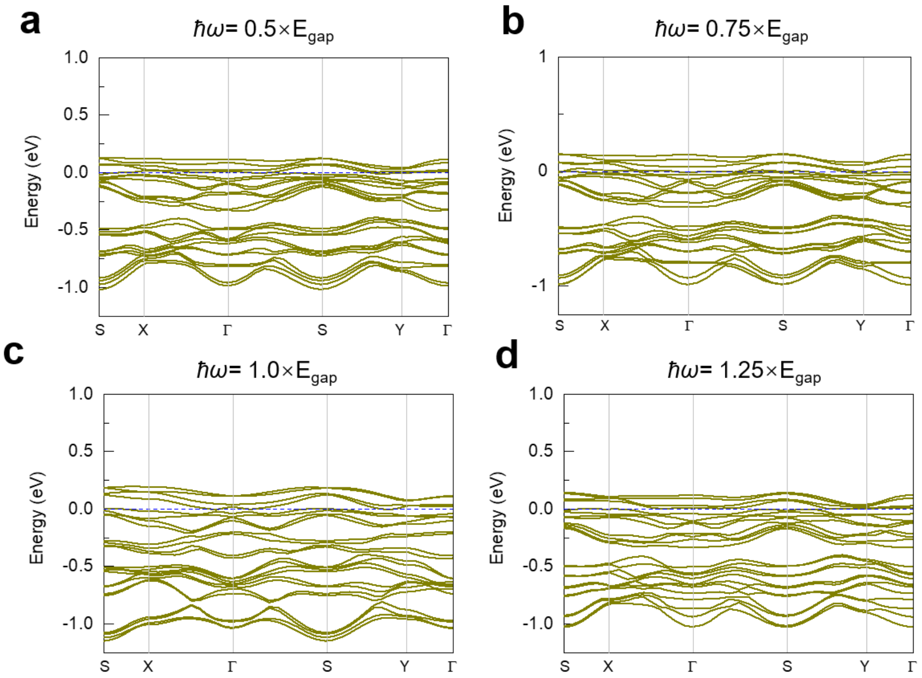

Figure S8. Time-dependent band structures with various photon energies. Band structure of α-$RuCl_3$ after photoexcitation, for various photon energy at 25 fs after photoexcitation, respectively. We use $I_0=2.5\times10^{12}$ W/cm^2 and the polarization is perpendicular to the magnetic moments. In all the cases, we observed (not shown here), that the Mott gap melts completely in less 20 fs.

The ultrafast insulator-to-metal transition is robust for various photon energies at the laser with strong intensity. As for other photon energies, the ultrafast band renormalization takes place within 15 fs. This explains the smaller modulation of magnetic moments. Our results validate the bandgap of α-$RuCl_3$ can be easily modulated by optical excitation.

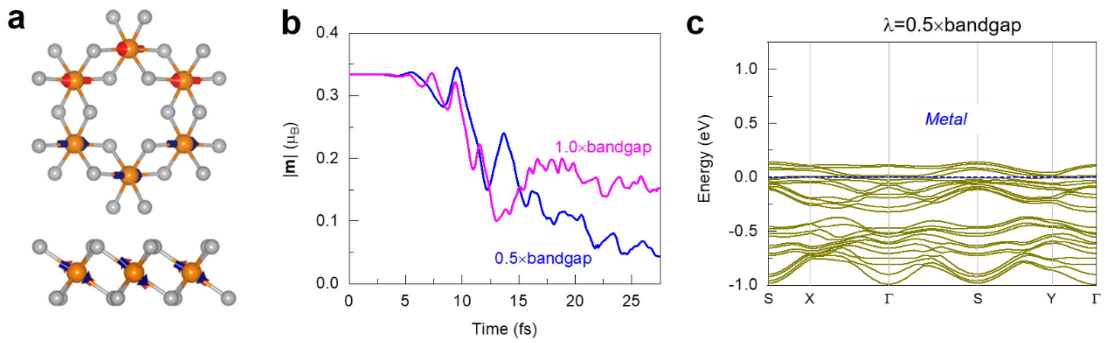

Figure S9. Laser-induced spin dynamics staring from a modulated zigzag magnetic order in α-RuCl3. (**a**) Atomic and magnetic structures of α-$RuCl_3$ with the modulated zigzag antiferromagnet order where the magnetic moments are oriented ±35° from the ab plane. (**b**) Dynamics of magnetic moment of four Ru atoms for laser pulses with parallel polarization and ħω= 0.5× and 1.0×E_{gap}, respectively. (**c**) Transient band structure of α-$RuCl_3$ after photoexcitation at 25.4 fs after photoexcitation, respectively. Here, we use I_0=0.5×10^{12} W/cm^2 and the polarization is perpendicular to the magnetic moments.

To validate the physical picture, we performed additional simulations from the modulated zigzag antiferromagnet order to track the magnetic dynamics and transient band structures. It is obvious that the laser-induced magnetic and electronic dynamics are similar for the two possible magnetic states (in-plane zigzag and modulated zigzag). Therefore, we obtain a robust picture of ultrafast photoinduced insulator-to-metal transition in α-$RuCl_3$.

Reference.

1. Andrade, X.; Strubbe, D.; De Giovannini, U.; Larsen, A. H.; Oliveira, M. J.; Alberti-Rodriguez, J.; Varas, A.; Theophilou, I.; Helbig, N.; Verstraete, M.; Stella, L.; Nogueira, F.; Aspuru-Guzik, A.; Castro, A.; Marques, M.; Rubio, A. Real-space grids and the Octopus code as tools for the development of new simulation approaches for electronic systems. *Phys. Chem. Chem. Phys*. **2015**, 17, 31371–31396.
2. Tancogne-Dejean, N.; Oliveira, M.J.; Andrade, X.; Appel, H.; Borca, C. H.; Le Breton, G.; Buchholz, F.; Castro, A.; Corni, S.; Correa, A. et al, Octopus, a computational framework for exploring light-driven phenomena and quantum dynamics in extended and finite systems. *J. Chem. Phys.* **2020**, 152 124119.

3. Banerjee, A.; Bridges, C.; Yan, J.-Q.; Aczel, A.; Li, L.; Stone, M.; Granroth, G.; Lumsden, M.; Yiu, Y.; Knolle, J. Proximate Kitaev quantum spin liquid behaviour in a honeycomb magnet. *Nat. Mat.* **2016**, 15, 733-740.
4. Agapito, L. A.; Curtarolo, S.; Nardelli, M. Reformulation of DFT+U as a pseudohybrid Hubbard density functional for accelerated materials discovery. *Phys. Rev. X* **2015**, 5, 011006.
5. Tancogne-Dejean, N.; Oliveira, M. J.; Rubio, A. Self-consistent DFT+U method for real-space time-dependent density functional theory calculations. *Phys. Rev. B* **2017**, 96, 245133.
6. Tancogne-Dejean, N.; Sentef, M. A.; Rubio, A. Ultrafast modification of Hubbard U in a strongly correlated material: ab initio high-harmonic generation in NiO. *Phys. Rev. Lett.* **2018**, 121, 097402.
7. Topp, G. E.; Tancogne-Dejean, N.; Kemper, A. F.; Rubio, A.; Sentef, M. All-optical nonequilibrium pathway to stabilising magnetic Weyl semimetals in pyrochlore iridates. *Nat. Commun.* **2018**, 9, 4452.
8. Kresse, G. *et al*, Ab initio molecular dynamics for liquid metals. *Phys. Rev. B* **1993,** 47, 558.
9. Kresse, G. ; Furthmüller, J.; Efficient iterative schemes for ab initio total-energy calculations using a plane-wave basis set. *Phys. Rev. B* **1996**, 54 (16), 11169−11186.
10. Perdew, J.; Burke, K.; Ernzerhof, M. Generalized gradient approximation made simple. *Phys. Rev. Lett.* **1996**, 77, 3865.
11. Tang, W.; Sanville, E.; Henkelman, G. A grid-based Bader analysis algorithm without lattice bias, *J. Phys.: Condens. Matter* **2009,** 21, 084204.
